# Editorials

**Published:** 1898-11

**Authors:** 


### EDITORIAL.

### AN IMPORTANT ACTION.

THE veterinary profession all over the land will watch with the keenest interest the progress and outcome of the lawsuit for damages entered upon for libellous statements by one of New York's prominent veterinarians. The misdoings of a few members of the profession in that city, through the taking of bribes, commissions, and other compensation than their legitimate fees in conducting examinations for soundness, have for many years been a serious shadow upon the general reputation of the profession. The writer has heard many dealers speak in scathing words of the demands of veterinarians of Gotham in conducting their sales, and indulging in such remarks as "There is not one of them that will not take a commission." Growing bolder in their general charges of dishonesty against the profession, one of them has intimated in writing to a would-be purchaser that he could not meet the fee demanded by his veterinarian, hence the rejection of his horses in the examination. In defence of the honor of the greater number of the profession a suit for $10,000 damages has been instituted against this dealer, and its successful termination will put an end to these imputations, very unjust to the great body of veterinarians of Greater New York, whose honesty and integrity in this trying work of their calling merit the fullest confidence of horse-buyers.

### IN 1899.

BALTIMORE has entered the field for the place of meeting of the A. V. M. A. next year. The State Society, through its President, has formally invited the Association to that Southern city, so famed for its open-hearted generosity and hospitality. One of the most successful meetings of the Association was held in the "Oriole City" in 1888, and its work, its reception, its influence for good were well marked, and added much to the general advancement and enhanced recognition of the profession. The devotion to Association interests of a number of Baltimore's veterinarians is of such a character as to make that city an attractive one, and would, had she not been so late in the field for the coveted honor, no doubt have found a place in the front rank.

Detroit, with her ever-zealous interest for the welfare and fame of the "City of Straits," is also knocking at the doors of the executive committee. The civic bodies, commercial organizations, etc., are ever anxious to bring to their city organized bodies and conventions of every character and kind. Her railroad facilities are all that could be desired, but her geographical position will hardly command the Association's meeting until Cleveland, Columbus, or some more central point is touched that will reach a larger number of those in that territory who should be active in the American association.

---

### TURN ON THE LIGHT.

THE controversy now going on between the Cattle Commissions of New Hampshire and Maine is destined to shed a great deal of light on the value of the several methods adopted in the various States of this country in dealing with the subject of tuberculosis among cattle. To scientific observers no more dangerous statements have been promulgated anywhere in the world than have emanated from the authorities in New Hampshire; based upon unfair or unscientific data or experiment; contrary to the conclusions drawn in many tens of thousands of cases, and in opposition to the expressed conclusions of the greatest authorities over the world. More than that, the most widespread publicity was given to these false deductions and conclusions; by farm and livestock journals all over the land, just at a time when an aroused public was startled at the knowledge of the extent of bovine tuberculosis, when livestock owners should have been foremost in supporting every movement for the extermination of this plague, as a matter of self-preservation, and be found supporting this work, but who, more eager to save a penny and waste a dollar, have checked a wise and intelligent disposition to eliminate and control this source of the disease by governmental assistance with a public sentiment indorsing the same. No State will reap the whirlwind more than New Hampshire, a distinctly dairy State, whose products, ever seeking a wider field of sale, will feel the force of deliberately maintaining within her herds the most dangerous infectious or contagious disease among bovines, a disease now known to affect almost every species, and the "white plague" of the whole earth.

---

Dr. Elmer Heaton Judkins, of New Paltz, N. Y., was married to Miss Jennie D. Auchmoody, of Highland, N. Y., on Wednesday, October 5th, at the residence of the bride's brother. Dr. Judkins is a graduate of the Ontario Veterinary College, class of '96.

